# Prediction of α-synuclein seed amplification assay positivity in remotely followed *LRRK2* G2019S carriers using a validated data-driven model

**DOI:** 10.1017/cts.2026.10756

**Published:** 2026-05-29

**Authors:** Ruth B. Schneider, Peggy Auinger, Charles S. Venuto

**Affiliations:** University of Rochester, Rochester, NY, USA

**Keywords:** α-synuclein, LRRK2, decentralized research, predict, Parkinson’s disease

## Abstract

The α-synuclein seed amplification assay (SAA) is a biomarker for Parkinson’s disease (PD) but requires cerebrospinal fluid. We applied a validated model to predict α-synuclein SAA+ using remotely collected data in LRRK2 G2019S carriers. VALOR-PD participants completed at-home olfactory and autonomic assessments. SAA+ probabilities for manifest and non-manifest carriers at baseline and month 36 visits were estimated. Baseline SAA+ mean probability was 0.57 (manifest) and 0.28 (non-manifest). Over three years, SAA+ mean probabilities increased in manifest (0.66, p = 0.014) and non-manifest carriers (0.38, p < 0.001), paralleling olfactory declines. Remotely collected data may be used to approximate SAA classification.

## Introduction

The cerebrospinal fluid (CSF) α-synuclein seed amplification assay (SAA) has emerged as a biomarker for Parkinson’s disease (PD) with α-synuclein status serving as the foundation for recently introduced PD biological classification and staging systems [1, 2]. In the near future, these developments will change the way PD clinical trials are conducted. For example, α-synuclein SAA status may be used to ensure that α-synuclein-targeting therapeutics are only tested in participants with evidence of pathological α-synuclein.

However, reliance on an assay that is collected by an invasive procedure that can only be performed in-clinic by a specialist poses some challenges. Implementation of widespread α-synuclein SAA testing in CSF will be limited by location (e.g., resource-limited and rural areas) and medical status (e.g., contraindications to lumbar puncture). While pathological α-synuclein can also be detected in skin [3, 4], and efforts are underway to develop less-invasive detection methods, there may be alternative approaches.

Recently, a data-driven model for predicting CSF α-synuclein SAA status was introduced [5]. The model was developed using data from the Parkinson Progression Marker Initiative (PPMI) and validated externally using data from the Systemic Synuclein Sampling Study. The final model, which incorporated smell test performance, sex, constipation, and genetic status (*GBA1* and *LRRK2*), was able to predict CSF α-synuclein SAA status with a high level of accuracy. Here, we apply the model in a remote, nationwide cohort of *LRRK2* G2019S carriers with and without PD in which CSF α-synuclein SAA status would not be available as part of the study design [6, 7].

## Methods

Data were obtained from VALOR-PD, a remote, prospective, nationwide, natural history study of *LRRK2* G2019S carriers with annual video visits accompanied by at-home smell assessments at baseline and month 36 [6]. VALOR-PD was conducted in accordance with the Declaration of Helsinki and approved by the Institutional Review Board of the University of Rochester (STUDY00003703). Informed consent was obtained from all study participants. The sample included both individuals with manifest PD and non-manifest carriers as determined by expert-trained investigators. To enable application of a previously published model for predicting CSF α-synuclein SAA positivity, relevant demographic, clinical, and genetic variables were harmonized [5]. These included age, sex, constipation symptoms (measured using the Scale for Outcomes in PD-Autonomic Dysfunction [SCOPA-AUT5] constipation item) [8], and olfactory function. Olfactory performance was assessed using the original version of the University of Pennsylvania Smell Identification Test (UPSIT). Age-and sex-adjusted percentile scores were derived from raw UPSIT scores following methods previously described [9]. GBA mutation status was not available in this dataset and was imputed as negative (i.e., non-carrier) for all participants, based on the low prevalence of individuals with both LRRK2 and GBA variants (∼2–5%) [5, 10].

The logistic regression model previously developed in the PPMI cohort was applied to VALOR-PD participants. The model estimates the probability of α-synuclein SAA positivity using the predictors: age-and sex-adjusted UPSIT percentile, sex, SCOPA-AUT5 constipation score, *LRRK2* variant type, and GBA status. Model coefficients come from the bootstrapped fully adjusted model. Predicted probabilities of α-synuclein SAA positivity were calculated for each participant at both the baseline and month 36 visits in VALOR-PD. The inverse logit function was applied to convert the linear predictor into a predicted probability. Because observed SAA status was not available in VALOR-PD, analyses focus primarily on the continuous predicted probabilities. For descriptive classification only, probabilities were also summarized using two pre-specified thresholds for classifying participants as predicted SAA positive (SAA+) or negative (SAA-): a conventional threshold of 0.5, and the previously derived Youden cut-off from the original model development cohort (0.778). Paired t-tests were used to evaluate changes in variables from baseline to month 36. Data processing and analysis were conducted in R version 4.3.1.

## Results

Among the 277 VALOR-PD participants, 254 had complete data at baseline (21 were excluded for incomplete UPSITs and 2 for missing SCOPA-AUT5). Of the 248 participants who completed their month 36 visit, 177 had complete data at that visit (71 were excluded for incomplete UPSITs). A total of 170 participants had complete data at both baseline and month 36 and were included in the longitudinal analysis. Table 1 summarizes baseline and month 36 characteristics and model-predicted SAA positivity probabilities by expert-assessed disease status for these 170 participants with longitudinal data (corresponding summaries for all participants regardless of availability of longitudinal follow-up are shown in Table S1).

**Table 1.** Summary characteristics for participants with both baseline and month 36 data by Parkinson’s disease diagnosis status determined at baseline* Table 1 long description.

	All (*n* = 170)	Non-manifest carriers (*n* = 128)	Manifest carriers (*n* = 42)
Age, years			
Baseline	59.8 (13.8)	56.8 (14.2)	68.9 (6.4)
Month 36	62.8 (13.8)	59.8 (14.3)	71.9 (6.4)
Female, *n* (%)	105 (62%)	83 (65%)	22 (52%)
Male, *n* (%)	65 (38%)	45 (35%)	20 (48%)
Race			
White	166 (97.6%)	125 (97.7%)	41 (97.6%)
Prefer not to answer	4 (2.4%)	3 (2.3%)	1 (2.4%)
Ethnicity			
Hispanic/Latino	7 (4.1%)	4 (3.1%)	3 (7.1%)
Not Hispanic/Latino	155 (91.2%)	117 (91.4%)	38 (90.5%)
Prefer not to answer	8 (4.7%)	7 (5.5%)	1 (2.4%)
Scale for outcomes in PD-autonomic dysfunction constipation item (SCOPA-AUT5)			
Baseline	0.33 (0.69)	0.21 (0.56)	0.69 (0.92)
Month 36	0.44 (0.78)	0.25 (0.52)	1.0 (1.10)
University of Pennsylvania Smell Identification Test (UPSIT) total correct			
Baseline	31.6 (6.6)	33.6 (4.7)	25.8 (8.0)
Month 36	29.7 (7.2)	31.9 (5.4)	23.0 (7.9)
UPSIT %ile			
Baseline	47.8 (30.7)	53.6 (29.0)	30.1 (29.3)
Month 36	38.4 (29.9)	43.5 (29.4)	22.8 (26.0)
Seed amplification assay (SAA+) probability			
Baseline	0.35 (0.33)	0.28 (0.29)	0.57 (0.34)
Month 36	0.45 (0.34)	0.38 (0.31)	0.66 (0.32)
SAA+ probability >0.5, *n* (%)			
Baseline	59 (34.7%)	33 (25.8%)	26 (61.9%)
Month 36	80 (47.1%)	49 (38.3%)	31 (73.8%)
SAA+ probability >0.778, *n* (%)			
Baseline	31 (18.2%)	12 (9.4%)	19 (45.2%)
Month 36	48 (28.2%)	23 (18.0%)	25 (59.5%)

*Data summarized as mean (standard deviation) unless otherwise indicated.

**Figure 1.**
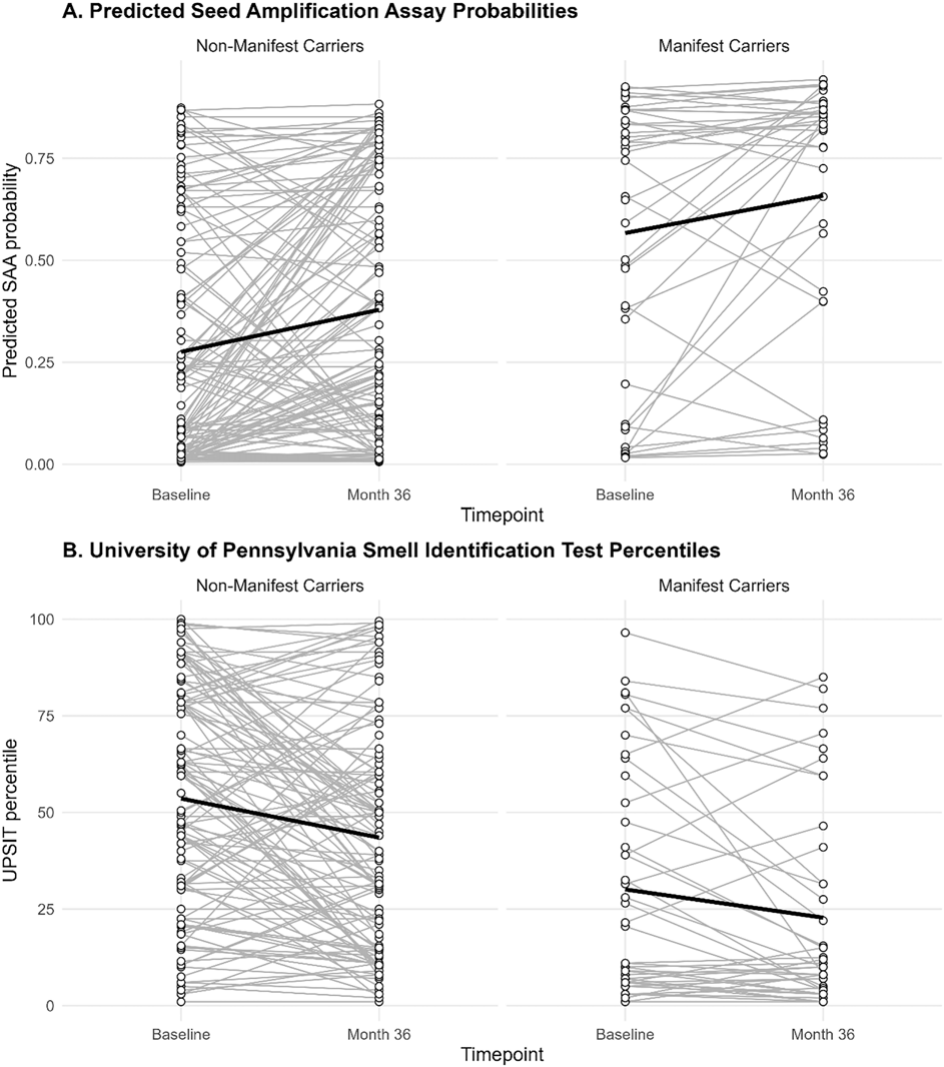
(A) Predicted cerebrospinal fluid α-synuclein SAA positivity probability at baseline and month 36 by Parkinson’s disease (PD) diagnosis status. Each gray line represents an individual’s predicted probability at baseline and month 36. (B) Age-and sex-adjusted University of Pennsylvania Smell Identification Test (UPSIT) percentile at baseline and month 36 by PD diagnosis status. Each gray line represents an individual’s UPSIT percentile at baseline and month 36. The dark black lines in each figure show the mean trend across all participants within each diagnosis group.

Among non-manifest carriers, those with longitudinal data (*n* = 128) were older at baseline than those with baseline-only data (*n* = 65) (mean [SD] age 56.8 [14.2] vs. 50.2 [14.8], *p* = 0.0037), but did not differ significantly on other baseline characteristics (Table S2). The longitudinal data of the non-manifest group showed the mean probability of being SAA+ significantly increased from 0.28 to 0.38 over the three-year follow-up (mean difference: 0.10 [paired *t*-test *p*-value = 0.000048]) (Figure 1A). The increase coincided with a decline in mean UPSIT age-and sex-adjusted percentile (53.6 to 43.5; mean difference:-10.1 [*p*-value = 0.000038]) (Figure 1B). There was not a significant change in mean SCOPA-AUT5 (0.21 to 0.25; mean difference: 0.039 [*p*-value = 0.47]).

Among manifest carriers, 42 participants had complete data at both baseline and month 36. There were no statistically significant differences in baseline characteristics between those with baseline-only data (*n* = 19) and those with longitudinal data (Table S3). The longitudinal data for the manifest group showed the overall mean probability of being SAA+ significantly increased from 0.57 to 0.66 (mean difference: 0.092 [paired *t*-test *p*-value = 0.014]) (Figure 1A). Participants also showed a significant decline in mean UPSIT percentile (30.1 to 22.8, mean difference: −7.30 [*p*-value = 0.016]) (Figure 1B), and a non-significant increase in mean SCOPA-AUT5 (0.69 to 1.00; mean difference: 0.31 [*p*-value = 0.051]).

## Discussion

In this study, we applied a previously validated data-driven model to a fully remote, nationwide cohort of *LRRK2* G2019S carriers participating in the VALOR-PD study. Using remotely collected demographics, olfactory, and autonomic data, we estimated the probability of CSF α-synuclein SAA positivity among individuals with and without manifest PD at baseline and month 36. Across time points, the distribution of predicted probabilities differed by clinical status, with higher probabilities observed among manifest compared with non-manifest *LRRK2* G2019S carriers. In addition, mean predicted SAA probability increased from baseline to Month 36 within both groups, although there was considerable within-person variability with individual trajectories showing both increases and decreases over time. These longitudinal changes reflect a shift in the model-estimated predicted rate of SAA positivity (rather than a change in the intrinsic performance of the model) based on changes in the input of individuals’ clinical features (e.g., worsening olfactory function, increase in constipation symptoms).

For descriptive threshold-based summaries, the proportion classified as predicted SAA+ varied by the selected cut-off. Using a threshold of 0.5, 67% of manifest carriers and 25% of non-manifest carriers were predicted SAA+, whereas using the previously published Youden threshold (0.778), approximately 47% of manifest carriers and 10% of non-manifest carriers were predicted SAA+. These manifest estimates of SAA positivity fall within previously reported ranges for *LRRK2* G2019S carriers (38.5–67.5%), whereas the non-manifest estimates are broadly comparable to, but slightly extend above, previously reported ranges (0–19%) [11–13]. These findings underscore that binary predicted SAA+ percentages are threshold-dependent and should be interpreted cautiously, particularly in the absence of observed SAA outcomes in VALOR-PD. Taken together, these comparisons support the utility of the model for probability-based risk stratification while also highlighting the importance of cohort composition, threshold selection, and potential calibration differences when applying the model to new populations.

In the prior modeling work, olfactory dysfunction, as measured by the UPSIT, was the strongest predictor of CSF α-synuclein SAA-positive status [5]. Baseline olfactory performance among manifest carriers in VALOR-PD was consistent with findings from the multi-site *LRRK2* Ashkenazi Jewish Consortium. Specifically, the VALOR-PD mean UPSIT score (25.0 ± 7.9) and percentile (27.0 ± 26.9) were comparable to those reported from the *LRRK2* Ashkenazi Jewish Consortium (24.2 ± 8.8; percentile 25.6 ± 28.4) [14]. Notably, lower olfactory performance in *LRRK2* PD is associated with earlier disease onset and faster motor decline, suggesting that olfaction provides a window into disease heterogeneity and progression. Together, these data highlight that olfaction could provide a scalable marker of PD heterogeneity and progression, even when assessed remotely.

The ability to estimate SAA-related probability status using remotely collected clinical data has several implications. First, it demonstrates the feasibility of estimating SAA-related biological probability to entirely remote, decentralized cohorts, an important advance for large-scale screening studies where lumbar puncture collection is impractical. Decentralized studies enable the conduct of research outside of the traditional research setting through, for example, the use of video visits, electronic patient-reported outcome measures, and accelerometer-based sensors [15]. The ability to apply SAA-related biological probability to remote, decentralized cohorts has the potential to expand clinical trial screening to individuals who might otherwise not be able to participate due to geography or disability. Second, this modeling approach could enable identification of putative SAA+ individuals in other remote or in-person genetic cohorts, providing a cost-effective and noninvasive strategy for clinical trial enrichment. For example, selecting participants based on high predicted probability of SAA positivity could substantially improve the efficiency of synuclein-targeting therapeutic trials by pre-screening those most likely to harbor underlying synuclein pathology. This approach could also aid in the efficient identification of putative SAA-positive individuals for trials that use the neuronal α-synuclein disease biological framework for defining disease.

Several limitations warrant consideration. First, the true CSF α-synuclein SAA status of participants in VALOR-PD is unknown; therefore, the model-based predictions represent probabilistic inferences rather than direct measurements. Second, missing UPSIT data reduced the number of evaluable participants, which could introduce bias if non-completion was related to disease status or other unmeasured factors. Third, the model assumed all participants were *GBA* non-carriers, though dual mutation carriers, while rare, may differ in SAA positivity rates.

It is also important to note that the model applied to VALOR-PD was derived in a heterogeneous population that included individuals with and without PD, idiopathic PD, and multiple genetic subgroups (e.g., different *LRRK2* variants and *GBA* carriers), which differs from the VALOR-PD cohort composed entirely of *LRRK2* G2019S carriers. This is particularly relevant because *LRRK2*-associated parkinsonism is neuropathologically heterogeneous and not all carriers with PD have Lewy-type pathology [16–18]. Accordingly, this variation in underlying Lewy-type pathology is an important consideration when interpreting SAA predicted positivity in *LRRK2*-enriched cohorts. Supporting the biological relevance of α-synuclein SAA in this setting, one small neuropathology-based study reported that α-synuclein real-time quaking-induced conversion in postmortem brain tissue and CSF correctly identified *LRRK2*-PD cases with Lewy-type pathology as positive, while cases without Lewy-type pathology were negative [18]. Therefore, future work should prioritize development and calibration of SAA prediction models specifically within LRRK2 populations with direct SAA measurements, and when available, pathology-correlated validation.

In summary, we demonstrate that a validated predictive model for CSF α-synuclein SAA positivity can be applied successfully to a remote, genetically defined cohort, reproducing the distribution of SAA positivity observed in manifest carriers and aligning with in-person olfactory performance metrics from other LRRK2 PD studies. These findings support the use of remote behavioral and clinical data to approximate biological classification in PD, offering a promising framework for large-scale screening and trial recruitment in synucleinopathies.

## Supporting information

10.1017/cts.2026.10756.sm001 Schneider et al. supplementary material Schneider et al. supplementary material

## Supplementary material

The supplementary material for this article can be found at https://doi.org/10.1017/cts.2026.10756.

## Table 1. Long description

The table presents data for 170 participants with complete baseline and month 36 data, divided into all participants, non-manifest carriers, and manifest carriers. It includes rows for age, gender, race, ethnicity, SCOPA-AUT5 scores, UPSIT total correct, UPSIT percentile, SAA+ probability, SAA+ probability >0.5, and SAA+ probability >0.778. Each row provides values for baseline and month 36. For example, the age at baseline is 59.8 years for all participants, 56.8 years for non-manifest carriers, and 68.9 years for manifest carriers. The table also includes percentages for gender, race, and ethnicity, as well as scores and probabilities for various tests and conditions.

Navigate back to Table 1..
