# Supplementary material for: Prediction of α-synuclein seed amplification assay positivity in remotely followed LRRK2 G2019S carriers using a validated data-driven model
Source: J Clin Transl Sci. 2026 May 29;10(1):e101. doi: 10.1017/cts.2026.10756 (PMC13312348; doi:10.1017/cts.2026.10756)
Supplement: Schneider et al. supplementary material [file S2059866126107560sup001.docx]

| Table S1. Summary Characteristics at Baseline and Month 36 for Participants Regardless of Having Longitudinal Data | | | | | | |
| --- | --- | --- | --- | --- | --- | --- |
|  | BASELINE | | | MONTH 36 | | |
|  | All  (n=254) | Non-Manifest Carriers  (n=193) | Manifest Carriers  (n=61) | All  (n=177)^1^ | Non-Manifest Carriers  (n=133) | Manifest Carriers  (n=43) |
| Age, years | 57.9 (14.7) | 54.5 (14.7) | 68.5 (8.0) | 62.7 (14.0) | 59.7 (14.4) | 72.0 (6.5) |
| Female, n (%)  Male, n (%) | 149 (59%)  105 (41%) | 118 (61%)  75 (39%) | 31 (51%)  30 (49%) | 111 (63%)  66 (37%) | 87 (65%)  46 (35%) | 23 (53%)  20 (47%) |
| Race  White  Asian  Prefer not to answer | 246 (96.9%)  2 (0.8%)  6 (2.4%) | 187 (96.9%)  1 (0.5%)  5 (2.6%) | 59 (96.7%)  1 (1.6%)  1 (1.6%) | 172 (97.2%)  0  5 (2.8%) | 128 (96.2%)  0  5 (3.8%) | 43 (100%)  0  0 |
| Ethnicity  Hispanic/Latino  Not Hispanic/Latino  Prefer not to answer | 18 (7.1%)  226 (89.0%)  10 (3.9%) | 14 (7.3%)  171 (88.6%)  8 (4.1%) | 4 (6.6%)  55 (90.2%)  2 (3.3%) | 7 (4.0%)  160 (90.4%)  10 (5.6%) | 4 (3.0%)  120 (90.2%)  9 (6.8%) | 3 (7.0%)  39 (90.7%)  1 (2.3%) |
| SCOPA-AUT5 | 0.29 (0.63) | 0.20 (0.52) | 0.57 (0.85) | 0.44 (0.77) | 0.26 (0.53) | 0.98 (1.1) |
| UPSIT total correct | 31.6 (6.7) | 33.7 (4.6) | 25.0 (7.9) | 29.9 (7.1) | 31.85 (5.5) | 23.6 (7.9) |
| UPSIT %ile | 47.3 (30.7) | 53.8 (29.0) | 27.0 (26.9) | 39.0 (29.8) | 43.9 (29.6) | 24.1 (25.8) |
| SAA+ Modeled Probability | 0.35 (0.33) | 0.27 (0.29) | 0.59 (0.32) | 0.44 (0.33) | 0.37 (0.31) | 0.64 (0.33) |
| SAA+ Probability >0.5, n (%) | 89 (35.0%) | 48 (24.9%) | 41 (67.2%) | 81 (45.8%) | 51 (38.3%) | 30 (69.8%) |
| SAA+ Probability >0.778, n (%) | 49 (19.3%) | 20 (10.4%) | 29 (47.5%) | 48 (27.1%) | 24 (18.0%) | 24 (55.8%) |
| ^1^Includes one (n=1) participant whose month 36 diagnosis was unknown.  Data summarized as mean (standard deviation) unless otherwise indicated.  SCOPA-AUT5 – Scale for Outcomes in PD-Autonomic Dysfunction constipation item; UPSIT – University of Pennsylvania Smell Identification Test; SAA – seeding amplification assay for alpha-synuclein | | | | | | |

| Table S2. Comparison of baseline characteristics between non-manifest carriers with baseline-only data vs baseline + month 36 data. | | | |
| --- | --- | --- | --- |
|  | Baseline Data Only  (n=65) | Baseline + Month 36 Data  (n=128) | P-value |
| Age, years | 50.2 (14.8) | 56.8 (14.2) | **0.0037** |
| Male, n (%) | 30 (46.2%) | 45 (35.2%) | 0.19 |
| SCOPA-AUT5 | 0.18 (0.43) | 0.21 (0.56) | 0.72 |
| UPSIT total correct | 33.9 (4.6) | 33.6 (4.7) | 0.59 |
| UPSIT%ile | 54.1 (29.2) | 53.6 (29.0) | 0.90 |
| SAA+ Modeled Probability | 0.27 (0.29) | 0.28 (0.29) | 0.93 |
| SAA+ Probability >0.5, n (%) | 15 (23.1%) | 33 (25.8%) | 0.81 |
| SAA+ Probability >0.778, n (%) | 8 (12.3%) | 12 (9.4%) | 0.70 |
| Data summarized as mean (standard deviation) unless otherwise indicated.  P-values come from Welch two-sample t-tests or Pearson’s chi-squared test. | | | |

| Table S3. Comparison of baseline characteristics between manifest carriers with baseline-only data vs baseline + month 36 data. | | | |
| --- | --- | --- | --- |
|  | Baseline Data Only  (n=19) | Baseline + Month 36 Data  (n=42) | P-value |
| Age, years | 67.7 (10.9) | 68.9 (6.4) | 0.65 |
| Male, n (%) | 10 (52.6%) | 20 (47.6%) | 0.93 |
| SCOPA-AUT5 | 0.32 (0.58) | 0.69 (0.92) | 0.061 |
| UPSIT total correct | 23.2 (7.7) | 25.8 (8.0) | 0.24 |
| UPSIT%ile | 20.3 (19.6) | 30.1 (29.3) | 0.13 |
| SAA+ Modeled Probability | 0.65 (0.26) | 0.57 (0.34) | 0.28 |
| SAA+ Probability >0.5, n (%) | 15 (78.9%) | 26 (61.9%) | 0.31 |
| SAA+ Probability >0.778, n (%) | 10 (52.6%) | 19 (45.2%) | 0.80 |
| Data summarized as mean (standard deviation) unless otherwise indicated.  P-values come from Welch two-sample t-tests or Pearson’s chi-squared test. | | | |
